# Iron Deficiency in Endocrine Diseases and the Therapeutic Role of Liposomal Iron: A Comprehensive Review

**DOI:** 10.3390/biomedicines14091944

**Published:** 2026-08-29

**Authors:** Sandro La Vignera, Rosita A. Condorelli

**Affiliations:** Department of Clinical and Experimental Medicine, University of Catania, 95123 Catania, Italy

**Keywords:** iron deficiency, endocrine disorders, thyroid function, diabetes mellitus, obesity, polyendocrine metabolic ovarian syndrome, hypogonadism, liposomal iron, sucrosomial iron, hepcidin

## Abstract

Iron deficiency (ID) represents one of the most prevalent nutritional disorders worldwide, affecting approximately 1.2 billion individuals. Beyond its well-established hematological consequences, emerging evidence demonstrates that ID profoundly impacts endocrine function across multiple organ systems. Iron serves as an essential cofactor for numerous enzymes involved in hormone synthesis, metabolism, and signaling pathways. This comprehensive review examines the bidirectional relationships between iron deficiency and endocrine pathologies, including thyroid disorders (hypothyroidism, Hashimoto’s thyroiditis, thyroid peroxidase impairment), diabetes mellitus (types 1 and 2), obesity, Polyendocrine Metabolic Ovarian Syndrome (PMOS), male hypogonadism, adrenal insufficiency, growth hormone deficiency, pituitary dysfunction, and parathyroid disorders. We systematically analyze the molecular mechanisms linking iron metabolism to endocrine dysfunction, with particular emphasis on thyroid peroxidase activity, insulin secretion and sensitivity, hepcidin regulation, testosterone synthesis, and cortisol production. Furthermore, we critically evaluate the therapeutic potential of liposomal iron supplementation, a novel delivery system that offers superior bioavailability and gastrointestinal tolerability compared to conventional oral iron formulations. Randomized controlled trials demonstrate that liposomal iron achieves comparable efficacy to intravenous iron while significantly reducing adverse events. This review synthesizes current evidence to provide clinicians with a comprehensive understanding of iron-endocrine interactions and evidence-based recommendations for iron supplementation in endocrine disorders. Recognition of these complex relationships is essential for optimizing diagnostic and therapeutic approaches in patients with concurrent iron deficiency and endocrine dysfunction.

## 1. Introduction

Iron deficiency (ID) and iron deficiency anemia (IDA) constitute the most common nutritional deficiencies globally, affecting approximately 1.2 billion people and disproportionately impacting women of reproductive age, children, and individuals with chronic diseases. While the hematological manifestations of iron deficiency are well characterized, accumulating evidence reveals that iron plays critical roles far beyond erythropoiesis, particularly in endocrine physiology and hormone metabolism.

Iron serves as an essential cofactor for numerous enzymes involved in cellular respiration, DNA synthesis, and critically, hormone synthesis and metabolism. The recognition that iron deficiency can profoundly impact endocrine function has given rise to the emerging field of “ferrocrinology”—the study of iron’s role in endocrine physiology [1]. This bidirectional relationship between iron status and endocrine function has significant clinical implications, as iron deficiency may exacerbate endocrine disorders, while certain endocrine pathologies can predispose to iron deficiency through various mechanisms.

The thyroid gland represents a particularly iron-sensitive endocrine organ, as thyroid peroxidase (TPO), the rate-limiting enzyme in thyroid hormone synthesis, is an iron-dependent hemoprotein [2]. Consequently, iron deficiency can impair thyroid hormone production and metabolism, potentially contributing to hypothyroidism or reducing the efficacy of levothyroxine replacement therapy [3]. Similarly, iron plays crucial roles in pancreatic β-cell function and insulin synthesis, with both iron deficiency and iron overload associated with impaired glucose metabolism [4,5].

In obesity, a paradoxical relationship exists wherein adipose tissue expansion and chronic low-grade inflammation lead to increased hepcidin production, resulting in functional iron deficiency despite adequate or even excessive iron stores [6,7]. This phenomenon has important implications for understanding the metabolic complications of obesity, including insulin resistance and type 2 diabetes mellitus. Furthermore, iron deficiency has been implicated in reproductive endocrine disorders, including polyendocrine metabolic ovarian syndrome (PMOS) in women [8] and hypogonadism in men [9], affecting fertility and sexual function.

Traditional oral iron supplementation is available in multiple formulations, including ferrous compounds (ferrous sulfate, ferrous fumarate, ferrous gluconate) and ferric compounds (ferric ammonium citrate, iron polymaltose complex), all with broadly comparable efficacy but variable individual tolerability. Ferrous sulfate, among the most commonly prescribed forms, is frequently associated with significant gastrointestinal adverse effects, including nausea, constipation, and abdominal discomfort, leading to poor adherence rates of 30–50% [10]. Intravenous iron formulations offer superior efficacy but carry risks of infusion reactions, require healthcare facility administration, and incur higher costs. Notably, iron III-carboxymaltose has been associated with clinically significant hypophosphatemia and, in some cases, osteomalacia; similar events have been reported less frequently with iron III-sucrose and iron III-derisomaltose, and some experts explicitly advise caution with carboxymaltose use in at-risk patients. Liposomal iron represents a novel third-generation oral iron delivery system that encapsulates iron within a phospholipid bilayer, protecting it from gastric degradation while enhancing intestinal absorption through direct enterocyte uptake [11,12]. Randomized controlled trials have demonstrated that liposomal iron formulations achieve hemoglobin and ferritin increases comparable to intravenous iron while maintaining the convenience and safety profile superior to conventional oral iron [11,13,14].

This comprehensive review aims to: (1) systematically examine the molecular and clinical evidence linking iron deficiency to major endocrine pathologies across all endocrine organ systems; (2) elucidate the pathophysiological mechanisms underlying these associations; (3) critically evaluate the clinical evidence for liposomal iron supplementation as a therapeutic option; and (4) provide evidence-based recommendations for clinicians managing patients with concurrent iron deficiency and endocrine disorders. Understanding these complex iron-endocrine interactions is essential for optimizing diagnostic accuracy, therapeutic efficacy, and patient outcomes in this increasingly recognized clinical scenario.

### Search Strategy

A PRISMA-ScR (Preferred Reporting Items for Systematic reviews and Meta-Analyses extension for Scoping Reviews)-aligned search was conducted across PubMed/MEDLINE, Scopus, and Web of Science (last search date: April 2025). Search terms included combinations of ‘iron deficiency’, ‘iron supplementation’, ‘liposomal iron’, ‘sucrosomial iron’, and individual endocrine conditions (‘thyroid disorders’, ‘hypothyroidism’, ‘Hashimoto’, ‘diabetes mellitus’, ‘polyendocrine metabolic ovarian syndrome’, ‘PMOS’, ‘hypogonadism’, ‘adrenal insufficiency’, ‘parathyroid disorders’, ‘pituitary dysfunction’, ‘pancreatic exocrine insufficiency’). Inclusion criteria encompassed original research articles, systematic reviews, and meta-analyses published in English from 2000 to 2025 in peer-reviewed journals. Studies focused exclusively on iron overload states, hereditary hemochromatosis, or non-iron micronutrient deficiencies without co-reporting iron status were excluded. Case reports and animal studies were included when human clinical data were limited, as noted in the relevant sections.

## 2. Iron Metabolism and Endocrine Function: Fundamental Concepts

Iron homeostasis is tightly regulated through a complex interplay of absorption, storage, utilization, and recycling mechanisms. Dietary iron exists in two forms: heme iron (from animal sources) and non-heme iron (from plant sources and fortified foods). Non-heme iron absorption is regulated by the divalent metal transporter 1 (DMT1) following reduction by duodenal cytochrome b (Dcytb), while heme iron is absorbed via heme carrier protein 1 (HCP1). Ferroportin, the sole known cellular iron exporter, transports iron from enterocytes and macrophages into circulation, where it binds to transferrin for delivery to tissues [15].

Hepcidin, a 25-amino acid peptide hormone synthesized primarily by hepatocytes, serves as the master regulator of systemic iron homeostasis. Hepcidin binds to ferroportin, inducing its internalization and degradation, thereby reducing iron absorption from the intestine and iron release from macrophages and hepatocytes [16]. Hepcidin expression is upregulated by iron overload and inflammation (via IL-6 signaling) and downregulated by iron deficiency, erythropoietic drive, and hypoxia. This regulatory system becomes dysregulated in various pathological states, including obesity-related chronic inflammation and certain endocrine disorders [6].

The concept of “ferrocrinology” emphasizes iron’s fundamental roles in endocrine physiology beyond its traditional hematological functions [1]. Iron-dependent enzymes are critical for multiple endocrine processes:

Iron’s endocrine roles operate across three hierarchical levels: (i) at the molecular/cellular level, iron serves as a cofactor for key enzymatic reactions, including thyroid peroxidase-dependent thyroid hormone synthesis, steroidogenic cytochrome P450 enzymes, and insulin secretory granule biogenesis; (ii) at the organ/tissue level, iron availability modulates glandular function, mitochondrial oxidative metabolism, and hormone secretory capacity; and (iii) at the systemic/organismal level, systemic iron status determines endocrine axis activity through hepcidin-mediated regulation. This multi-level framework distinguishes ‘ferrocrinology’ from classical nutritional endocrinology, which focuses primarily on macro- and micronutrient substrates rather than cofactors integral to hormonal biosynthetic machinery.

A clinically important distinction must be drawn between nutritional iron deficiency—caused by inadequate dietary intake, reduced absorption, or chronic blood loss—and inflammation-driven functional iron deficiency, in which total body iron stores may be normal or elevated but cellular availability is reduced by hepcidin-mediated iron sequestration. In inflammatory states characteristic of obesity, type 2 diabetes, and autoimmune endocrinopathies, serum ferritin is an acute-phase reactant and may be elevated even in the presence of true cellular iron deficiency; conversely, low transferrin saturation (<20%) and elevated soluble transferrin receptor provide more reliable indicators of functional iron deficiency in these contexts. This distinction has direct therapeutic implications: nutritional iron deficiency responds to standard oral or intravenous supplementation, whereas functional iron deficiency requires concurrent treatment of the underlying inflammatory state alongside iron repletion.

- Thyroid hormone synthesis: Thyroid peroxidase (TPO), a heme-containing enzyme, catalyzes the iodination of thyroglobulin and the coupling of iodotyrosines to form T3 and T4 [2].
- Steroid hormone synthesis: Cytochrome P450 enzymes, which contain iron-sulfur clusters and heme groups, are essential for steroidogenesis in the adrenal cortex, gonads, and placenta [17].
- Insulin synthesis and secretion: Iron-sulfur cluster assembly proteins are required for proper mitochondrial function in pancreatic β-cells, and iron deficiency impairs insulin synthesis and glucose-stimulated insulin secretion [4].
- Catecholamine synthesis: Tyrosine hydroxylase, the rate-limiting enzyme in catecholamine biosynthesis, requires iron as a cofactor [18].
- Cellular energy metabolism: Iron-sulfur clusters and heme groups are integral components of the electron transport chain complexes, directly impacting ATP production in all endocrine tissues [19].

**Figure 1:**
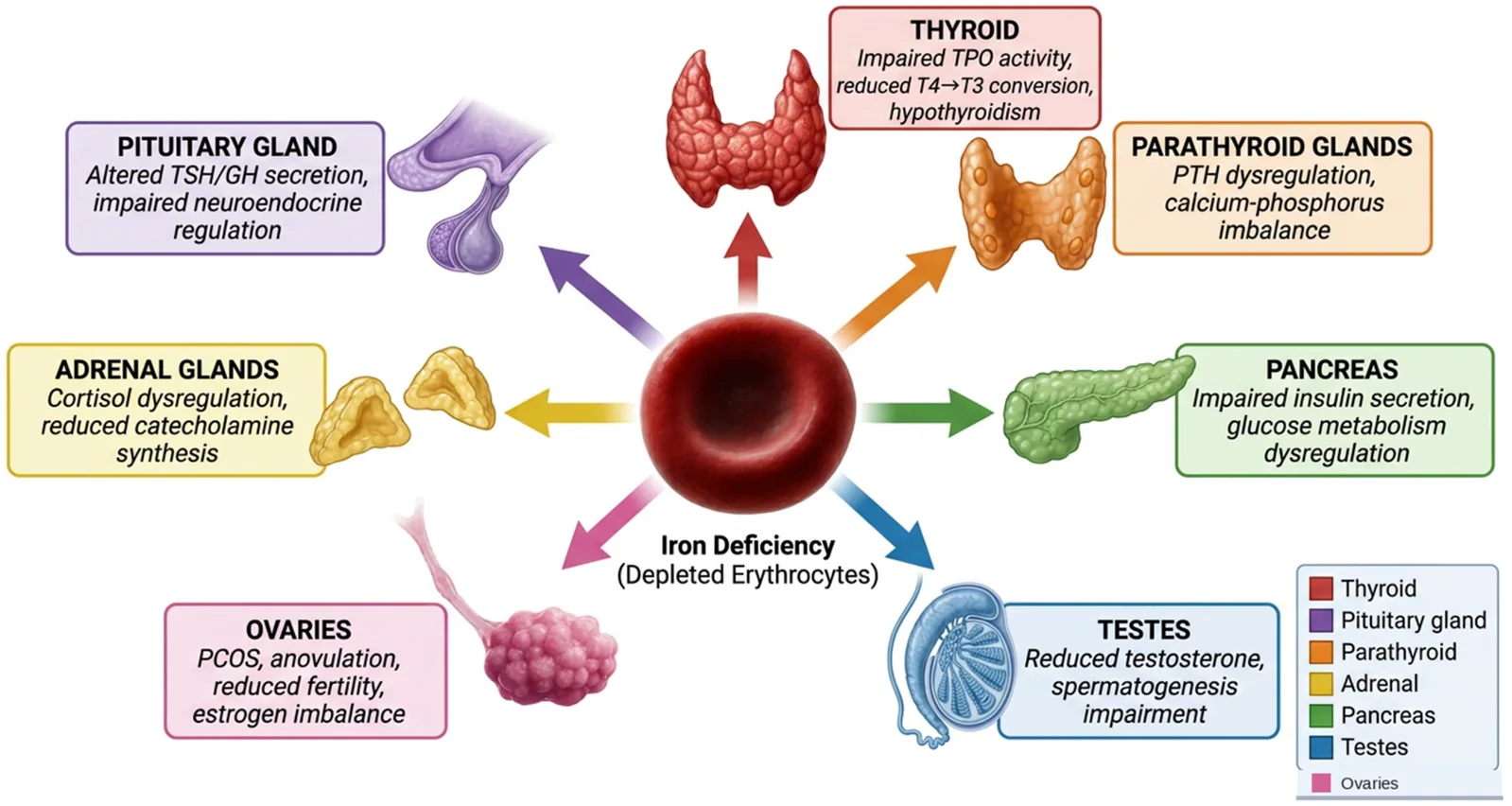
Schematic illustration of the seven major endocrine organs affected by iron deficiency (thyroid gland, pituitary gland, parathyroid glands, pancreas, testes, ovaries, and adrenal glands), demonstrating the systemic impact of iron on endocrine physiology. Colour-coded arrows connect the central iron-deficiency element (depleted erythrocytes) to each organ with its associated pathological conditions.

Iron deficiency can therefore impair endocrine function through multiple mechanisms: (1) reduced activity of iron-dependent enzymes involved in hormone synthesis; (2) impaired mitochondrial function and cellular energy metabolism; (3) altered gene expression through iron-responsive element (IRE) and iron-regulatory protein (IRP) systems; and (4) indirect effects through anemia-induced tissue hypoxia (Figure 1). Conversely, certain endocrine disorders can predispose to iron deficiency through mechanisms including increased hepcidin expression (obesity, insulin resistance), menstrual blood loss (PMOS, hyperprolactinemia), malabsorption (hypothyroidism), and altered iron metabolism (diabetes mellitus) [20].

Understanding these fundamental concepts is essential for appreciating the complex bidirectional relationships between iron status and endocrine function examined in the following sections.

## 3. Thyroid Disorders and Iron Deficiency

The thyroid gland exhibits particular sensitivity to iron status due to the critical dependence of thyroid hormone synthesis on thyroid peroxidase (TPO), an iron-containing hemoprotein. Accumulating evidence demonstrates bidirectional relationships between iron deficiency and thyroid dysfunction, with important clinical implications for diagnosis and management.

### 3.1. Hypothyroidism and Iron Deficiency Anemia

A systematic review and meta-analysis by Garofalo et al. [2] comprehensively examined the relationship between iron deficiency and thyroid function across ten cross-sectional studies. Patients with iron deficiency demonstrated significantly lower thyroid-stimulating hormone (TSH) levels (mean difference: −0.24 mIU/L, *p* = 0.005), free thyroxine (FT4) levels (mean difference: −1.18 pmol/L, *p* < 0.000001), and free triiodothyronine (FT3) levels (mean difference: −0.22 pmol/L, *p* < 0.00001) compared to iron-replete controls. Importantly, serum TSH and FT4 levels demonstrated positive correlations with serum ferritin concentrations, suggesting a dose-dependent relationship between iron status and thyroid function.

Subgroup analyses revealed that these associations persisted in both pregnant and non-pregnant women, although the magnitude of effect varied. In pregnant women, iron deficiency was associated with lower TSH, FT4, and FT3 levels, while non-pregnant women showed significant reductions in FT4 and FT3 but not TSH [2]. These findings suggest that iron deficiency may impair thyroid hormone synthesis and peripheral conversion of T4 to T3, potentially through reduced TPO activity and decreased hepatic 5′-deiodinase activity.

Islam et al. [21] conducted a community-based cross-sectional study of 452 anemic women in Bangladesh, revealing that 24.69% of women with iron deficiency anemia had concurrent hypothyroidism, with the highest prevalence (38.46%) observed in women aged 31–40 years. A strong correlation (*p* < 0.0001) was observed between serum iron levels and TSH concentrations. Notably, among children aged 0–14 years with congenital hypothyroidism, 50% also had iron deficiency anemia, suggesting that early-life iron deficiency may contribute to thyroid dysfunction [21].

The clinical implications of these findings are significant. Szczepanek-Parulska et al. [22] noted that anemia occurs in 20–60% of patients with overt hypothyroidism and 10–15% of those with subclinical hypothyroidism. The mechanisms underlying hypothyroidism-associated anemia are multifactorial and include: (1) reduced erythropoietin production due to decreased metabolic rate; (2) impaired iron absorption secondary to reduced gastric acid secretion; (3) menorrhagia related to anovulatory cycles; and (4) potential autoimmune gastritis in patients with autoimmune thyroid disease [22].

### 3.2. Hashimoto’s Thyroiditis and Autoimmunity

Iron deficiency has been implicated in the pathogenesis and progression of autoimmune thyroid disease, particularly Hashimoto’s thyroiditis. The meta-analysis by Garofalo et al. [2] demonstrated that iron deficiency was associated with increased prevalence of thyroid autoantibody positivity, including both anti-thyroglobulin (anti-Tg) and anti-thyroid peroxidase (anti-TPO) antibodies.

The mechanisms linking iron deficiency to thyroid autoimmunity are complex and multifactorial. Iron is essential for proper immune function, and iron deficiency can lead to dysregulation of both innate and adaptive immunity. Specifically, iron deficiency may: (1) impair T-regulatory cell function, reducing immune tolerance; (2) alter the balance between Th1 and Th2 responses; (3) increase oxidative stress, potentially exposing cryptic thyroid antigens; and (4) directly affect thyroid follicular cell function, potentially increasing antigen presentation [23].

Islam et al. [21] specifically noted that iron deficiency in early pregnancy can lead to hypothyroidism due to autoimmunity, explained by increased antibodies against thyroid peroxidase enzymes (TPOAb). This observation suggests that adequate iron status during critical developmental periods may be important for preventing autoimmune thyroid disease.

The clinical relevance of this association is underscored by the observation that iron supplementation may improve thyroid function in patients with subclinical hypothyroidism and concurrent iron deficiency. Ravanbod et al. [3] demonstrated that treatment of iron deficiency anemia in patients with subclinical hypothyroidism led to improvements in thyroid function parameters, suggesting that iron repletion should be considered as part of the therapeutic approach in these patients.

### 3.3. Thyroid Peroxidase Impairment

Thyroid peroxidase (TPO) is a membrane-bound hemoprotein localized to the apical surface of thyroid follicular cells, where it catalyzes three critical reactions in thyroid hormone synthesis: (1) oxidation of iodide to iodine; (2) iodination of tyrosyl residues in thyroglobulin to form monoiodotyrosine (MIT) and diiodotyrosine (DIT); and (3) coupling of iodotyrosines to form T3 and T4 [24]. As a heme-containing enzyme, TPO activity is directly dependent on adequate iron availability.

Gökdeniz et al. [25] investigated the effects of iron deficiency anemia on thyroid function and identified both secondary and subclinical hypothyroidism in iron-deficient patients. The key pathophysiological mechanisms identified included: (1) reduced thyroid peroxidase activity due to insufficient iron for heme synthesis; (2) impaired peripheral conversion of T4 to T3, possibly due to reduced hepatic 5′-monodeiodinase activity; and (3) altered hypothalamic-pituitary-thyroid axis regulation. Importantly, these hormonal abnormalities normalized following iron supplementation, demonstrating the reversibility of iron deficiency-induced thyroid dysfunction [25].

The molecular basis for TPO impairment in iron deficiency involves multiple mechanisms. First, heme biosynthesis requires iron as a substrate for ferrochelatase, the enzyme that inserts ferrous iron into protoporphyrin IX to form heme. Iron deficiency therefore directly limits heme availability for TPO synthesis [26]. Second, iron deficiency may impair the post-translational processing and trafficking of TPO to the apical membrane, reducing the amount of functional enzyme at the site of hormone synthesis [27]. Third, iron deficiency-induced oxidative stress may damage TPO protein structure and function [28].

Dahiya et al. [29] examined the mutual relationship between thyroid profile and iron metabolism in hypothyroidism, demonstrating bidirectional effects. Not only does iron deficiency impair thyroid function, but hypothyroidism itself can contribute to iron deficiency through: (1) reduced gastric acid secretion, impairing non-heme iron absorption; (2) decreased intestinal motility, potentially affecting iron absorption; (3) menstrual irregularities leading to increased blood loss; and (4) reduced expression of iron transport proteins [29].

The clinical implications of TPO impairment in iron deficiency are significant for thyroid disease management. Patients with concurrent iron deficiency and hypothyroidism may exhibit: (1) suboptimal response to levothyroxine replacement therapy; (2) persistently elevated TSH despite adequate levothyroxine dosing; (3) symptoms of hypothyroidism despite biochemically euthyroid status; and (4) increased risk of progression from subclinical to overt hypothyroidism [3]. These observations support the recommendation that iron status should be assessed and corrected in all patients with thyroid dysfunction, particularly those with suboptimal treatment response.

## 4. Diabetes Mellitus and Iron Metabolism

The relationship between iron metabolism and diabetes mellitus is complex and bidirectional, with both iron deficiency and iron overload implicated in the pathogenesis and complications of diabetes. Iron plays critical roles in pancreatic β-cell function, insulin synthesis, and glucose metabolism, while diabetes-related complications can alter iron homeostasis.

### 4.1. Type 1 Diabetes Mellitus

Type 1 diabetes mellitus (T1DM) is characterized by autoimmune destruction of pancreatic β-cells, leading to absolute insulin deficiency. While the primary pathophysiology involves immune-mediated β-cell destruction, iron metabolism abnormalities may contribute to disease progression and complications.

Iron is essential for proper β-cell function through multiple mechanisms. Mitochondrial iron-sulfur cluster assembly is required for oxidative phosphorylation and ATP production, which drives glucose-stimulated insulin secretion [4]. Additionally, iron-dependent enzymes are involved in insulin synthesis, processing, and secretion. Iron deficiency may therefore exacerbate β-cell dysfunction in the early stages of T1DM or during periods of partial β-cell recovery (the “honeymoon period”).

Ramesh et al. [4] examined the impact of iron indices on insulin secretion and demonstrated that iron deficiency impairs mitochondrial oxidative capacity in β-cells, reducing glucose-stimulated insulin secretion. This effect is mediated through reduced activity of iron-sulfur cluster-containing enzymes in the electron transport chain, leading to decreased ATP production and impaired closure of ATP-sensitive potassium channels, which is essential for insulin granule exocytosis [4].

Furthermore, iron deficiency in T1DM patients may complicate glycemic control through effects on erythrocyte lifespan and hemoglobin glycation. Iron deficiency prolongs erythrocyte survival, potentially leading to falsely elevated HbA1c values that do not accurately reflect average glycemia [30]. This phenomenon has important implications for diabetes management and will be discussed in detail in Section 4.3.

### 4.2. Type 2 Diabetes Mellitus and Insulin Resistance

Type 2 diabetes mellitus (T2DM) is characterized by insulin resistance and progressive β-cell dysfunction. The relationship between iron metabolism and T2DM is particularly complex, as both iron deficiency and iron excess have been implicated in disease pathogenesis.

Fernández-Real et al. [5] were among the first to describe the “cross-talk between iron metabolism and diabetes,” demonstrating that elevated body iron stores, as reflected by increased serum ferritin concentrations, are associated with increased risk of T2DM. This association appears to be mediated through iron-induced oxidative stress, which impairs insulin signaling in peripheral tissues and promotes β-cell dysfunction [5]. Conversely, iron deficiency may also contribute to insulin resistance through impaired mitochondrial function and reduced oxidative capacity in skeletal muscle and adipose tissue.

The concept of “ferrocrinology” as applied to glucose and lipid metabolism has been comprehensively reviewed by Szklarz et al. [1]. Iron deficiency can lead to obesity and T2DM through several mechanisms: (1) impaired exercise capacity due to reduced oxygen delivery and mitochondrial dysfunction; (2) reduced AMP-activated protein kinase (AMPK) activity, a key regulator of cellular energy homeostasis; (3) enhanced insulin resistance through impaired insulin receptor signaling; (4) dysfunction of brown adipose tissue due to impaired browning and reduced mitochondrial iron content; and (5) impaired thermogenesis via reduced thyroid hormone binding to nuclear receptors [1].

Abichandani [31] recently reviewed the role of iron metabolism in obesity, T2DM, atherosclerotic cardiovascular disease, and metabolic dysfunction-associated steatotic liver disease, emphasizing the complex relationships between iron status and metabolic health. The review highlighted that iron deficiency in the context of obesity and metabolic syndrome often represents functional iron deficiency due to hepcidin-mediated iron sequestration rather than absolute iron depletion [31].

Mascitelli et al. [32] examined hyperinsulinemia and iron perturbation in patients with T2DM, demonstrating that insulin resistance is associated with altered iron metabolism characterized by elevated serum ferritin but reduced serum iron and transferrin saturation. This pattern suggests iron sequestration in storage sites (hepatocytes, macrophages) with reduced iron availability for erythropoiesis and other metabolic functions [32]. The mechanisms underlying this phenomenon involve insulin-mediated upregulation of ferritin synthesis and inflammation-induced hepcidin production.

### 4.3. Iron Deficiency and HbA1c Interpretation

Hemoglobin A1c (HbA1c) is the gold standard biomarker for assessing long-term glycemic control in diabetes mellitus, reflecting average blood glucose concentrations over the preceding 2–3 months. However, HbA1c values can be significantly affected by conditions that alter erythrocyte lifespan or hemoglobin glycation rates, including iron deficiency anemia.

Christy et al. [30] investigated the influence of iron deficiency anemia on HbA1c levels in diabetic individuals with controlled plasma glucose levels. The study demonstrated that iron deficiency anemia was associated with significantly elevated HbA1c values compared to non-anemic diabetic patients with similar fasting and postprandial glucose levels. This discrepancy occurs because iron deficiency prolongs erythrocyte lifespan, allowing more time for hemoglobin glycation to occur, resulting in falsely elevated HbA1c values that overestimate true glycemic control [30].

Soliman et al. [33] comprehensively reviewed iron deficiency anemia and glucose metabolism, emphasizing that the relationship between iron status and HbA1c has important clinical implications. In patients with iron deficiency anemia, HbA1c may not accurately reflect glycemic control, potentially leading to: (1) overestimation of diabetes severity; (2) inappropriate intensification of glucose-lowering therapy; (3) increased risk of hypoglycemia; and (4) misclassification of diabetes control status [33].

The magnitude of this effect can be substantial. Studies have shown that HbA1c values may be elevated by 0.5–1.5% in patients with iron deficiency anemia compared to iron-replete individuals with identical mean glucose levels [30]. Following iron repletion and correction of anemia, HbA1c values typically decrease to levels more accurately reflecting true glycemic control.

These findings have important clinical implications for diabetes management:

- Diagnostic considerations: Iron deficiency should be excluded before using HbA1c for diabetes diagnosis, as falsely elevated values may lead to overdiagnosis.
- Treatment monitoring: In patients with concurrent diabetes and iron deficiency anemia, fasting and postprandial glucose measurements or continuous glucose monitoring may provide more accurate assessments of glycemic control than HbA1c.
- Treatment targets: Clinicians should be cautious about intensifying glucose-lowering therapy based solely on elevated HbA1c in patients with iron deficiency anemia, as this may increase hypoglycemia risk.
- Iron repletion: Correction of iron deficiency should be prioritized in diabetic patients with anemia, both to improve iron-dependent metabolic functions and to restore the accuracy of HbA1c as a glycemic biomarker.

## 5. Obesity and Iron Deficiency: The Hepcidin Connection

Obesity is paradoxically associated with increased risk of iron deficiency despite adequate or even excessive dietary iron intake. This phenomenon, termed “obesity-related iron deficiency,” is mediated primarily through chronic low-grade inflammation and dysregulated hepcidin expression, representing a form of functional iron deficiency similar to anemia of chronic disease.

### 5.1. Mechanisms of Obesity-Related Iron Deficiency

Cepeda-Lopez et al. [6] comprehensively reviewed whether obesity increases risk for iron deficiency, identifying multiple mechanisms underlying this association. The primary mechanism involves obesity-associated chronic inflammation, characterized by elevated circulating levels of pro-inflammatory cytokines, particularly interleukin-6 (IL-6) and tumor necrosis factor-alpha (TNF-α). These cytokines stimulate hepatic hepcidin synthesis, which in turn binds to ferroportin on enterocytes and macrophages, inducing its internalization and degradation [6]. This reduces both dietary iron absorption and iron release from body stores, leading to functional iron deficiency characterized by low serum iron and transferrin saturation despite normal or elevated ferritin levels.

Tussing-Humphreys et al. [7] emphasized the importance of “rethinking iron regulation and assessment in iron deficiency, anemia of chronic disease, and obesity” by introducing hepcidin as a central regulatory molecule. In obesity, adipose tissue itself becomes a source of inflammatory cytokines through macrophage infiltration and adipocyte dysfunction. Adipocytes can also directly produce hepcidin, contributing to systemic iron sequestration [7]. This adipocyte-derived hepcidin blocks the production of leptin and adiponectin, creating a vicious cycle that perpetuates metabolic dysfunction.

McClung et al. [34] examined iron deficiency and obesity, highlighting the contribution of inflammation and diminished iron absorption. The review noted that obese individuals demonstrate reduced iron absorption efficiency even when dietary iron intake is adequate, due to hepcidin-mediated downregulation of duodenal iron transporters, including divalent metal transporter 1 (DMT1) and ferroportin [34]. Additionally, obesity-related gastroesophageal reflux and increased use of proton pump inhibitors may further impair iron absorption by reducing gastric acidity.

Aigner et al. [35] characterized obesity as an emerging risk factor for iron deficiency, particularly in women of reproductive age and adolescents. The study demonstrated that the prevalence of iron deficiency increases with body mass index (BMI), with obese individuals having 2–3 times higher risk of iron deficiency compared to normal-weight individuals [35]. This association persists after adjusting for dietary iron intake, menstrual blood loss, and other confounding factors, supporting a causal role for obesity-related inflammation in iron deficiency pathogenesis.

### 5.2. Adipokines and Iron Metabolism

Fadem [36] explored the complex relationships between iron regulation, obesity, and anemia, with particular emphasis on the role of adipokines. Obesity interferes with iron regulation through multiple pathways involving adipocyte dysfunction. Macrophage recruitment into adipose tissue increases inflammatory cytokine production, which stimulates hepcidin release from both adipocytes and the liver. Hepcidin then blocks intestinal iron absorption and sequesters iron in adipocytes, which in turn blocks the production of leptin and adiponectin [36].

Leptin, an adipokine that regulates appetite and energy expenditure, requires adequate iron for its synthesis and secretion. Iron deficiency in obesity therefore creates a paradoxical situation where expanded adipose tissue mass is associated with reduced leptin production, potentially contributing to leptin resistance and further weight gain [36]. Similarly, adiponectin, which enhances insulin sensitivity and has anti-inflammatory properties, is reduced in iron-deficient obese individuals, potentially exacerbating insulin resistance and metabolic dysfunction.

### 5.3. Clinical Implications and Type 2 Diabetes Risk

The relationship between obesity-related iron deficiency and type 2 diabetes mellitus is particularly important. Szklarz et al. [1] demonstrated that iron deficiency can lead to obesity and T2DM by impairing exercise capacity, reducing AMPK activity, and enhancing insulin resistance. Iron deficiency also causes dysfunction of brown adipose tissue due to impaired browning and reduced mitochondrial iron content, impairing thermogenesis and energy expenditure [1].

Abichandani [31] comprehensively reviewed the role of iron metabolism in obesity, T2DM, atherosclerotic cardiovascular disease, and metabolic dysfunction-associated steatotic liver disease. The review emphasized that the relationship between iron and metabolic disease is U-shaped, with both iron deficiency and iron excess associated with adverse metabolic outcomes [31]. In obesity, the challenge is to distinguish between true iron deficiency requiring supplementation and functional iron deficiency due to inflammation, where iron supplementation may be less effective and potentially harmful.

### 5.4. Diagnostic and Therapeutic Considerations

The diagnosis of iron deficiency in obese individuals requires careful interpretation of iron parameters. Serum ferritin, the most commonly used marker of iron stores, is an acute-phase reactant that is elevated in inflammatory states, potentially masking concurrent iron deficiency [7]. In obese individuals, ferritin levels may be normal or elevated despite true iron depletion. Therefore, additional markers should be considered, including:

- Transferrin saturation: Values < 20% suggest iron deficiency even when ferritin is normal.
- Soluble transferrin receptor (sTfR): Elevated levels indicate tissue iron deficiency and are less affected by inflammation.
- sTfR/log ferritin ratio: This ratio helps distinguish iron deficiency from anemia of chronic disease.
- Hepcidin levels: Elevated hepcidin in the presence of low transferrin saturation suggests functional iron deficiency.

Treatment of iron deficiency in obesity presents unique challenges. Oral iron supplementation may be less effective due to hepcidin-mediated blockade of intestinal iron absorption [6,7]. Intravenous iron can bypass this blockade but may exacerbate oxidative stress and inflammation. Liposomal iron formulations, which are absorbed through alternative pathways less affected by hepcidin, may offer advantages in this population, as discussed in Section 11.

## 6. Polyendocrine Metabolic Ovarian Syndrome (PMOS) and Iron Deficiency

Polyendocrine metabolic ovarian syndrome (PMOS) is the most common endocrine disorder in women of reproductive age, affecting 5–15% of this population [37]. PMOS is characterized by hyperandrogenism, ovulatory dysfunction, and polycystic ovarian morphology, with associated metabolic complications including insulin resistance, obesity, and increased cardiovascular risk. Emerging evidence suggests bidirectional relationships between iron status and PMOS pathophysiology.

### 6.1. Iron Deficiency in PMOS: Prevalence and Mechanisms

Al-Akabi et al. [8] investigated the physiological effect of iron status on patients with PMOS in Basrah city, demonstrating significant alterations in iron parameters among PMOS patients compared to healthy controls. The study revealed that women with PMOS had lower serum iron levels, reduced transferrin saturation, and higher total iron-binding capacity (TIBC), indicating iron deficiency [8]. The prevalence of iron deficiency in PMOS patients was significantly higher than in age-matched controls, even after adjusting for BMI and menstrual blood loss.

Several mechanisms may contribute to increased iron deficiency risk in PMOS:

- Menstrual blood loss: Although PMOS is characterized by oligomenorrhea or amenorrhea, when menstruation occurs, it is often heavy and prolonged due to anovulatory cycles and endometrial hyperplasia, leading to increased iron loss [8].
- Chronic inflammation: PMOS is associated with chronic low-grade inflammation, characterized by elevated C-reactive protein (CRP) and pro-inflammatory cytokines. This inflammatory state may increase hepcidin production, leading to functional iron deficiency similar to that observed in obesity [38].
- Insulin resistance and metabolic dysfunction: Insulin resistance, present in 50–70% of PMOS patients, is associated with altered iron metabolism. Hyperinsulinemia may increase ferritin synthesis and iron sequestration while reducing iron availability for erythropoiesis [32].
- Obesity: Approximately 50–80% of PMOS patients are overweight or obese, and obesity-related iron deficiency (discussed in Section 5) contributes to overall iron deficiency risk in this population [35].

### 6.2. Iron Deficiency Effects on PMOS Pathophysiology

Iron deficiency may exacerbate several key features of PMOS:

Ovulatory dysfunction: Iron is essential for proper ovarian function, including folliculogenesis, ovulation, and corpus luteum function. Iron-dependent enzymes, including cytochrome P450 aromatase, are critical for estrogen synthesis in granulosa cells [39]. Iron deficiency may therefore impair follicular development and ovulation, worsening the anovulation characteristic of PMOS.

Hyperandrogenism: The relationship between iron status and androgen metabolism in PMOS is complex. Some evidence suggests that iron deficiency may affect androgen synthesis and metabolism through effects on cytochrome P450 enzymes involved in steroidogenesis [17]. However, the directionality and magnitude of this effect in PMOS patients requires further investigation.

Insulin resistance: As discussed in Section 4.2, iron deficiency can enhance insulin resistance through multiple mechanisms, including impaired mitochondrial function, reduced AMPK activity, and altered insulin signaling [1]. In PMOS patients, who already have high baseline insulin resistance, concurrent iron deficiency may further worsen metabolic dysfunction and increase risk of progression to type 2 diabetes.

Fertility outcomes: Iron deficiency has been associated with reduced fertility in women, potentially through effects on ovulation, endometrial receptivity, and early pregnancy maintenance [40]. In PMOS patients seeking fertility, iron deficiency may represent an additional modifiable barrier to conception.

### 6.3. Clinical Implications

The recognition of iron deficiency as a potential contributor to PMOS pathophysiology has several clinical implications:

- Screening: Iron status assessment (complete blood count, serum ferritin, transferrin saturation) should be considered in all PMOS patients, particularly those with menstrual irregularities, obesity, or metabolic complications.
- Interpretation: In PMOS patients with concurrent obesity and inflammation, ferritin levels should be interpreted cautiously, with consideration of additional markers such as transferrin saturation and soluble transferrin receptor.
- Treatment: Iron supplementation should be considered in PMOS patients with documented iron deficiency, with potential benefits for menstrual regularity, ovulation, metabolic function, and fertility outcomes.
- Formulation selection: Given the high prevalence of obesity and gastrointestinal symptoms in PMOS patients, liposomal iron formulations may offer advantages in terms of absorption efficiency and tolerability compared to conventional oral iron.

## 7. Male Hypogonadism and Reproductive Function

While iron deficiency’s effects on female reproductive function have received considerable attention, emerging evidence demonstrates that iron status also significantly impacts male reproductive endocrinology, including testosterone synthesis, spermatogenesis, and overall reproductive function.

### 7.1. Iron Deficiency and Testosterone Synthesis

Prasad et al. [9] described a syndrome of iron deficiency anemia, hepatosplenomegaly, hypogonadism, dwarfism, and geophagia in young men, representing one of the earliest recognitions of the relationship between iron deficiency and male hypogonadism. The study demonstrated that severe iron deficiency was associated with delayed sexual maturation, reduced testicular size, and low testosterone levels [9]. Importantly, iron supplementation led to improvements in growth, sexual development, and testosterone levels, demonstrating the reversibility of iron deficiency-induced hypogonadism.

The mechanisms linking iron deficiency to reduced testosterone synthesis involve multiple pathways:

- Leydig cell dysfunction: Testosterone synthesis in Leydig cells requires multiple cytochrome P450 enzymes, including CYP11A1 (cholesterol side-chain cleavage enzyme), CYP17A1 (17α-hydroxylase/17,20-lyase), and 17β-hydroxysteroid dehydrogenase. These enzymes contain iron-sulfur clusters and heme groups essential for their catalytic activity [17]. Iron deficiency therefore directly impairs the enzymatic machinery required for testosterone biosynthesis.
- Mitochondrial function: Testosterone synthesis is an energy-intensive process requiring adequate ATP production. Iron deficiency impairs mitochondrial oxidative phosphorylation through reduced activity of iron-sulfur cluster-containing complexes I, II, and III of the electron transport chain [19]. This energy deficit may limit testosterone synthesis capacity in Leydig cells.
- Hypothalamic-pituitary-gonadal (HPG) axis: Iron deficiency may affect the HPG axis at multiple levels, including gonadotropin-releasing hormone (GnRH) secretion from the hypothalamus and luteinizing hormone (LH) and follicle-stimulating hormone (FSH) secretion from the pituitary. Anemia-induced hypoxia may also affect HPG axis function [41].

### 7.2. Effects on Spermatogenesis and Sperm Quality

Iron is essential for spermatogenesis, the complex process of sperm production in the seminiferous tubules. Iron-dependent processes critical for spermatogenesis include:

- DNA synthesis: Ribonucleotide reductase, the rate-limiting enzyme in DNA synthesis, requires iron for its activity. Iron deficiency may therefore impair the rapid cell divisions characteristic of spermatogenesis [42].
- Mitochondrial function: Developing spermatocytes and spermatids have high energy requirements, and iron deficiency-induced mitochondrial dysfunction may impair sperm maturation and motility [19].
- Oxidative stress protection: Iron is a component of antioxidant enzymes including catalase and peroxidases. Iron deficiency may increase oxidative stress in the testicular environment, potentially damaging developing sperm and reducing sperm quality [43].
- Sertoli cell function: Sertoli cells provide structural and nutritional support for developing germ cells. Iron deficiency may impair Sertoli cell function, indirectly affecting spermatogenesis [44].

### 7.3. Clinical Implications in Thyroid Disorders

The relationship between iron deficiency and male hypogonadism has important clinical implications:

- Evaluation of hypogonadism: Iron status assessment should be considered in men presenting with symptoms of hypogonadism, particularly in the presence of anemia or risk factors for iron deficiency.
- Infertility evaluation: In couples undergoing infertility evaluation, male iron status should be assessed, as iron deficiency may contribute to reduced sperm quality and fertility potential.
- Treatment considerations: In men with concurrent iron deficiency and hypogonadism, iron repletion should be attempted before initiating testosterone replacement therapy, as iron supplementation alone may improve testosterone levels and symptoms in some cases [9].
- Monitoring: In men receiving testosterone replacement therapy, iron status should be monitored, as testosterone therapy can stimulate erythropoiesis and potentially deplete iron stores, particularly in men with marginal iron status at baseline.

## 8. Adrenal Insufficiency and Cortisol Metabolism

The adrenal glands synthesize multiple hormones essential for stress response, electrolyte balance, and metabolic regulation, including cortisol, aldosterone, and catecholamines. Iron plays critical roles in adrenal steroidogenesis and catecholamine synthesis, and iron deficiency may therefore impact adrenal function.

### 8.1. Iron and Cortisol Synthesis

Cortisol synthesis in the zona fasciculata of the adrenal cortex requires a series of enzymatic reactions catalyzed by cytochrome P450 enzymes, including CYP11A1, CYP17A1, CYP21A2, and CYP11B1. These enzymes contain heme groups and iron-sulfur clusters essential for their catalytic activity [17]. Iron deficiency may therefore impair cortisol synthesis capacity, potentially contributing to or exacerbating adrenal insufficiency.

Szklarz et al. [1] noted that iron deficiency impairs cortisol synthesis and distribution, making stress reactivity more pronounced. This observation suggests that iron-deficient individuals may have reduced cortisol reserve and impaired ability to mount appropriate cortisol responses to physiological stress [1]. The clinical implications of this phenomenon may include increased susceptibility to stress-related symptoms, impaired glucose counter-regulation during hypoglycemia, and reduced cardiovascular stress responses.

### 8.2. Catecholamine Synthesis

The adrenal medulla synthesizes catecholamines (epinephrine and norepinephrine) essential for the acute stress response. Tyrosine hydroxylase, the rate-limiting enzyme in catecholamine biosynthesis, is an iron-dependent enzyme that catalyzes the conversion of tyrosine to L-DOPA [18]. Iron deficiency may therefore reduce catecholamine synthesis capacity, potentially impairing the sympathoadrenal response to stress.

Additionally, dopamine β-hydroxylase, which converts dopamine to norepinephrine, is a copper-dependent enzyme, but its expression and activity may be indirectly affected by iron status through effects on cellular energy metabolism and gene expression [45].

### 8.3. Diagnostic and Therapeutic Considerations

Based on limited and largely hypothesis-level evidence, the following clinical scenarios have been proposed in which iron deficiency may contribute to impaired adrenal stress responses; robust clinical data are lacking and these considerations should be interpreted with caution:

- Subclinical adrenal dysfunction: Iron deficiency may unmask or exacerbate subclinical adrenal insufficiency, leading to symptoms of fatigue, orthostatic hypotension, and impaired stress tolerance.
- Critical illness: In critically ill patients with concurrent iron deficiency and stress, impaired cortisol synthesis may contribute to relative adrenal insufficiency and hemodynamic instability.
- Chronic fatigue: Iron deficiency-related impairment of cortisol and catecholamine synthesis may contribute to chronic fatigue symptoms, which may be partially reversible with iron repletion.
- Hypoglycemia risk: Impaired cortisol and catecholamine responses to hypoglycemia in iron-deficient diabetic patients may increase risk of severe hypoglycemia and impaired hypoglycemia awareness.

Further research is needed to fully characterize the clinical significance of iron deficiency’s effects on adrenal function and to determine whether iron supplementation improves adrenal function or stress responses in iron-deficient individuals.

## 9. Growth Hormone Deficiency and Pituitary Function

The pituitary gland, often termed the “master gland,” regulates multiple endocrine axes through secretion of tropic hormones including growth hormone (GH), thyroid-stimulating hormone (TSH), adrenocorticotropic hormone (ACTH), luteinizing hormone (LH), follicle-stimulating hormone (FSH), and prolactin. Iron deficiency may affect pituitary function through multiple mechanisms, with particular relevance to growth and development.

### 9.1. Iron Deficiency and Growth Impairment

Alkuwari et al. [46] investigated the impact of iron deficiency anemia on growth and endocrine functions, demonstrating reversibility with treatment. The study revealed that children with iron deficiency anemia exhibited growth retardation, delayed puberty, and alterations in multiple endocrine parameters [46]. Importantly, iron supplementation led to improvements in growth velocity, sexual maturation, and normalization of endocrine function, demonstrating the causal role of iron deficiency in these abnormalities.

The mechanisms linking iron deficiency to growth impairment are multifactorial:

- Growth hormone–IGF-1 axis: Iron is an essential cofactor for pituitary somatotroph cell function, GH synthesis, and downstream IGF-1 production. Iron deficiency impairs GH secretion from the pituitary gland, reduces hepatic IGF-1 synthesis, and attenuates IGF-1 receptor signalling in target tissues, collectively contributing to growth retardation and pubertal delay [46,47].
- Thyroid function: As discussed in Section 3, iron deficiency impairs thyroid hormone synthesis and metabolism. Since thyroid hormones are essential for normal growth and development, iron deficiency-induced hypothyroidism contributes to growth retardation [2,3].
- Energy metabolism: Growth is an energy-intensive process requiring adequate ATP production. Iron deficiency-induced mitochondrial dysfunction limits energy availability for anabolic processes, contributing to growth impairment [19].
- Appetite and nutrition: Iron deficiency is associated with reduced appetite and altered food preferences, potentially leading to inadequate caloric and protein intake, further impairing growth [48].

### 9.2. Pituitary Function and Iron Status

The pituitary gland has high metabolic activity and is therefore sensitive to iron deficiency’s effects on cellular energy metabolism. Iron deficiency may affect pituitary function through several mechanisms:

- Mitochondrial dysfunction: Pituitary cells have high energy requirements for hormone synthesis and secretion. Iron deficiency impairs mitochondrial oxidative phosphorylation, potentially reducing the capacity for hormone production [19].
- Hypothalamic regulation: Iron deficiency may affect hypothalamic neurons that regulate pituitary function through secretion of releasing and inhibiting hormones. Anemia-induced hypoxia may also alter hypothalamic-pituitary signaling [41].
- Oxidative stress: Iron deficiency paradoxically increases oxidative stress in some tissues through impaired antioxidant enzyme function. Oxidative stress may damage pituitary cells and impair their function [49].

### 9.3. Clinical Implications in Male Hypogonadism

The relationship between iron deficiency and pituitary function has important clinical implications:

- Growth evaluation: Children presenting with growth retardation should undergo comprehensive evaluation including iron status assessment. Iron deficiency should be corrected before attributing growth failure to GH deficiency or other endocrine causes.
- Pubertal delay: Adolescents with delayed puberty should be evaluated for iron deficiency, as iron repletion may restore normal pubertal progression in some cases [9,46].
- Pituitary function testing: Iron deficiency may affect the results of dynamic pituitary function tests. Ideally, iron deficiency should be corrected before performing stimulation tests for GH, ACTH, or gonadotropins to ensure accurate interpretation.
- Treatment monitoring: In children receiving treatment for endocrine disorders (e.g., GH replacement, thyroid hormone replacement), concurrent iron deficiency should be identified and treated to optimize treatment response.

## 10. Parathyroid Disorders and Calcium Metabolism

While the relationship between iron deficiency and parathyroid function has received less attention than other endocrine systems, emerging evidence suggests potential interactions between iron status and calcium-phosphate metabolism.

### 10.1. Iron and Parathyroid Hormone Synthesis

Parathyroid hormone (PTH) is synthesized by chief cells in the parathyroid glands and plays a central role in calcium homeostasis. PTH synthesis and secretion are primarily regulated by serum calcium levels through the calcium-sensing receptor (CaSR). While direct evidence for iron’s role in PTH synthesis is limited, several theoretical considerations suggest potential interactions:

- Mitochondrial function: PTH synthesis and secretion require adequate cellular energy production. Iron deficiency-induced mitochondrial dysfunction may impair the capacity for PTH synthesis, particularly during periods of increased demand (e.g., hypocalcemia) [19].
- Oxidative stress: The parathyroid glands are sensitive to oxidative stress, which can impair PTH secretion and parathyroid cell function. Iron deficiency may alter the balance between pro-oxidant and antioxidant systems, potentially affecting parathyroid function [49].
- Vitamin D metabolism: Iron is required for proper function of cytochrome P450 enzymes involved in vitamin D metabolism, including 25-hydroxylase (CYP2R1) and 1α-hydroxylase (CYP27B1). Iron deficiency may therefore impair vitamin D activation, leading to secondary hyperparathyroidism [50].

### 10.2. Calcium Absorption and Iron Status

Calcium and iron share some common absorption pathways in the intestine, and interactions between these minerals may affect their bioavailability:

- Competitive absorption: Calcium and iron compete for absorption through divalent metal transporter 1 (DMT1). High calcium intake may reduce iron absorption, while high iron intake may reduce calcium absorption [51].
- Gastric acid: Both calcium and iron absorption are enhanced by gastric acid. Conditions that reduce gastric acid secretion (e.g., hypothyroidism, proton pump inhibitor use) may impair absorption of both minerals [52].
- Vitamin D: Vitamin D enhances calcium absorption and may also affect iron metabolism through effects on hepcidin expression. Iron deficiency-induced impairment of vitamin D activation may therefore indirectly affect calcium homeostasis [50].

### 10.3. Clinical Considerations

**Figure 2:**
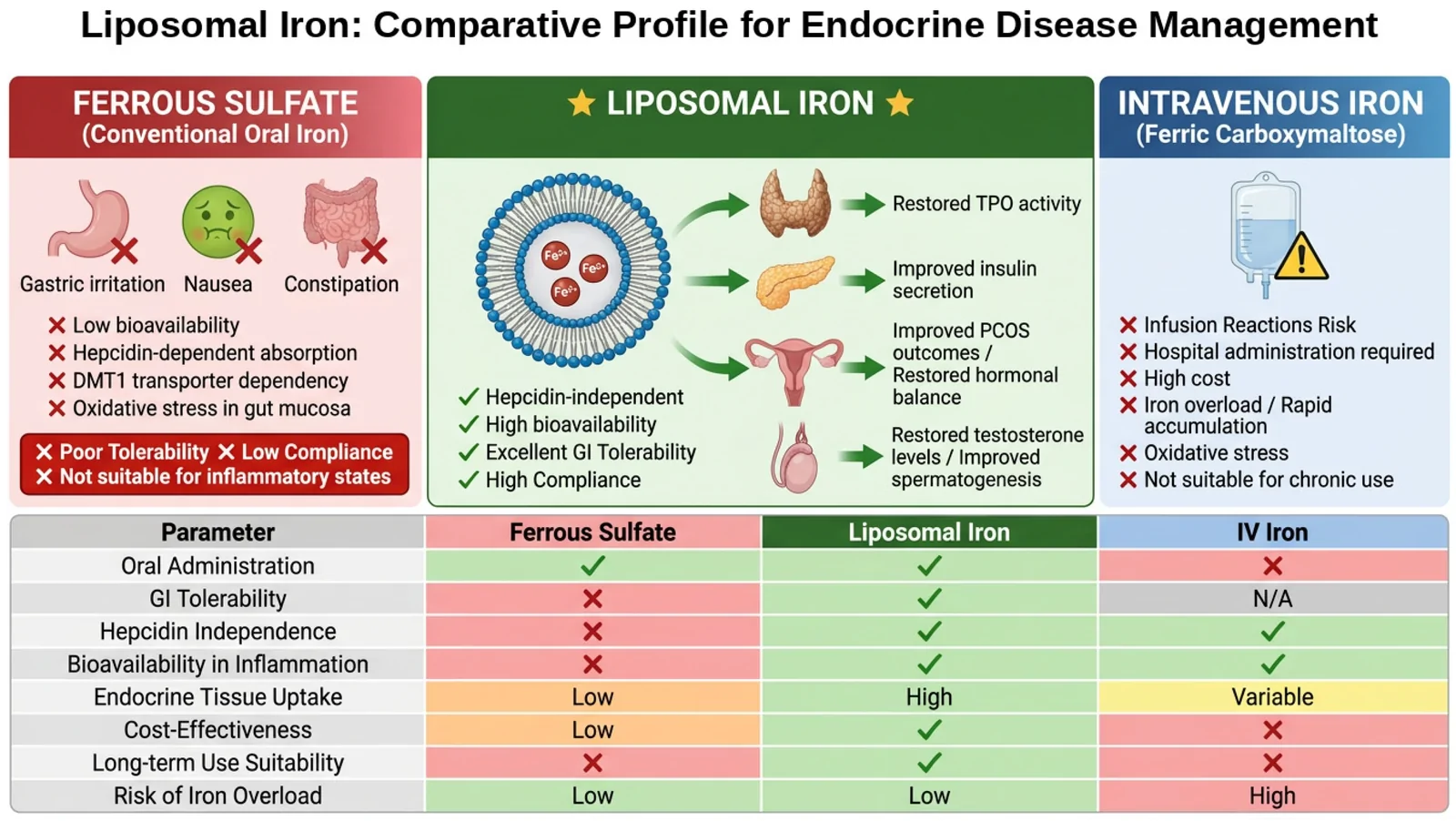
Comparative profile of liposomal iron versus ferrous sulfate (conventional oral iron) and intravenous iron (ferric carboxymaltose) for endocrine disease management. The central column highlights the favorable profile of liposomal iron: hepcidin-independent absorption, excellent gastrointestinal tolerability, high bioavailability in inflammatory states, and restored endocrine organ function (thyroid, pancreas, ovaries, testes). Bottom panel: summary comparison matrix.

Based on emerging and limited evidence, the clinical significance of iron-parathyroid interactions remains largely at the hypothesis level; the following practical considerations are proposed pending more robust clinical data (Figure 2):

- Supplementation timing: When both calcium and iron supplementation are required, they should be administered at different times of day to minimize competitive absorption.
- Secondary hyperparathyroidism: In patients with secondary hyperparathyroidism due to vitamin D deficiency, iron status should be assessed and corrected to ensure optimal vitamin D metabolism.
- Chronic kidney disease: Patients with chronic kidney disease often have concurrent iron deficiency, secondary hyperparathyroidism, and disordered mineral metabolism. Comprehensive management should address all of these abnormalities.
- Bone health: Both iron deficiency and parathyroid disorders affect bone health. Iron is required for collagen synthesis and osteoblast function, and iron deficiency may contribute to osteoporosis risk, particularly in postmenopausal women [53].

## 11. Liposomal Iron Supplementation: Mechanism and Clinical Evidence

Traditional oral iron supplementation with ferrous sulfate, while effective and inexpensive, is associated with significant gastrointestinal adverse effects that limit adherence and therapeutic efficacy. Intravenous iron formulations offer superior efficacy but require healthcare facility administration, carry risks of infusion reactions, and incur higher costs. Liposomal iron represents a novel third-generation oral iron delivery system that addresses many limitations of conventional formulations.

### 11.1. Mechanism of Action and Bioavailability

Liposomal iron formulations encapsulate iron (typically ferric pyrophosphate or ferric hydroxide) within a phospholipid bilayer vesicle, mimicking the structure of cell membranes [11]. This innovative delivery system offers several advantages:

Protection from gastric degradation: The phospholipid bilayer protects iron from gastric acid and digestive enzymes, preventing the formation of insoluble iron complexes and reducing gastric irritation [12]. This protection maintains iron in a form suitable for absorption throughout the gastrointestinal tract.

Enhanced intestinal absorption: Liposomal iron is absorbed through multiple mechanisms distinct from conventional iron salts. While ferrous sulfate requires reduction by duodenal cytochrome b (Dcytb) and transport via divalent metal transporter 1 (DMT1), liposomal iron can be absorbed through: (1) direct fusion of liposomes with enterocyte membranes; (2) endocytosis of intact liposomes; and (3) paracellular transport [11,12]. These alternative absorption pathways are less affected by hepcidin-mediated regulation, potentially offering advantages in inflammatory conditions characterized by elevated hepcidin levels.

Reduced hepcidin sensitivity: Maladkar et al. [12] emphasized that liposomal iron absorption is less dependent on ferroportin, the cellular iron exporter that is degraded by hepcidin. This characteristic makes liposomal iron particularly attractive for patients with functional iron deficiency due to inflammation, including those with obesity, chronic kidney disease, and inflammatory bowel disease [12].

Improved bioavailability: Multiple studies have demonstrated that liposomal iron achieves bioavailability comparable to or exceeding that of ferrous sulfate despite containing lower elemental iron doses. Tinsley et al. [54] conducted a randomized crossover trial demonstrating that liposomal iron significantly increased hemoglobin and ferritin levels compared to standard iron formulations [54].

### 11.2. Comparison with Conventional Iron Formulations

#### 11.2.1. Liposomal Iron Versus Ferrous Sulfate

Multiple randomized controlled trials have directly compared liposomal iron with ferrous sulfate, the most commonly prescribed oral iron formulation:

Monazah et al. [55] conducted a randomized controlled trial in infants aged 4–6 months with iron deficiency anemia, comparing liposomal iron drops (15 mg/mL elemental iron) with conventional ferrous sulfate drops (25 mg/mL elemental iron), both administered daily for six months. Liposomal iron resulted in significantly greater increases in hemoglobin (β = 0.35 at 2 months; β = 0.51 at 6 months; both *p* < 0.05) and mean corpuscular hemoglobin (MCH) compared to ferrous sulfate, despite containing lower elemental iron content [55]. Both groups showed similar effects on ferritin and mean corpuscular volume (MCV), but liposomal iron led to a more pronounced reduction in total iron-binding capacity (TIBC), suggesting more efficient iron utilization.

Rangaraj et al. [56] compared liposomal iron (1 mg/kg/day) with conventional elemental iron (ferrous ascorbate, 3 mg/kg/day) in children aged 6–60 months with iron deficiency anemia for one month. Ferrous ascorbate significantly increased mean hemoglobin to 9.3 g/dL versus 8.5 g/dL for liposomal iron (*p* = 0.0018), demonstrating superior short-term efficacy at the higher dose [56]. However, liposomal iron showed significantly better adherence (*p* = 0.047) and adequate oral tolerance (*p* = 0.042), with less constipation (*p* = 0.046). This study highlights the trade-off between maximal efficacy and tolerability, with liposomal iron offering advantages in real-world adherence.

Hussain et al. [57] evaluated the efficacy of a novel food supplement (Ferfer^®^) containing microencapsulated iron in liposomal form (14 mg iron per 1.5-gm sachet, with vitamin C and vitamin B12) in 558 women with iron deficiency anemia. After 12 weeks, mean serum hemoglobin increased from 8.71 ± 2.24 g/dL to 10.47 ± 1.69 g/dL (*p* < 0.001) [57]. The study noted enhanced palatability and higher bioavailability with reduced gastrointestinal side effects. The dropout rate was approximately 21.6% (437 of 558 completed), which is favorable compared to typical dropout rates of 30–50% with ferrous sulfate.

#### 11.2.2. Liposomal Iron Versus Intravenous Iron

Several studies have compared oral liposomal iron with intravenous iron formulations, particularly in chronic kidney disease patients:

Pisani et al. [11] conducted a landmark randomized trial comparing oral liposomal iron (30 mg/day) with intravenous iron gluconate (1000 mg total dose, administered as 125 mg weekly) for 3 months in non-dialysis chronic kidney disease patients with iron deficiency anemia. Short-term intravenous iron showed a more rapid hemoglobin increase, but the final hemoglobin increase was similar for both treatments [11]. Replenishment of iron stores was greater in the intravenous group. Importantly, hemoglobin remained stable post-withdrawal in the intravenous group but returned to baseline in the oral group, suggesting the need for maintenance oral therapy. Adverse events were significantly lower in the oral liposomal iron group (*p* < 0.001), and adherence was similar between groups.

Zahr et al. [13] conducted a non-inferiority study comparing oral liposomal iron (30 mg daily) with injectable iron sucrose (weekly for three months) in non-dialysis chronic kidney disease patients. Both treatments increased hemoglobin; the intravenous group showed a 14.65% increase (*p* = 0.049), and the oral group showed a 4.78% increase (*p* = 0.003) [13]. The intravenous group also had significant increases in ferritin (*p* < 0.001) and transferrin saturation (*p* = 0.031). Adverse reactions included hypotension in the intravenous group (30.7%) and constipation in the oral group (28.4%). The study concluded that oral liposomal iron was safe and effective, though less efficacious in replenishing iron stores than intravenous iron.

Koura et al. [14] demonstrated the dramatic effect of oral liposomal iron (30 mg/day for 3 months) versus intravenous iron dextran (50 mg three times/week for 3 months) on chronic kidney disease pediatric anemia in a randomized clinical trial. Oral liposomal iron showed higher efficacy than intravenous dextran, with significant improvements within the group for hemoglobin, ferritin, and other parameters after 3 months [14]. Both groups had no statistically significant difference between them for these markers. Notably, no side effects were reported for liposomal iron, leading to higher compliance, whereas intravenous iron carried typical infusion-related risks.

#### 11.2.3. Liposomal Iron Versus Other Oral Formulations

Mishra et al. [58] compared liposomal iron with ferrous ascorbate for treatment of iron deficiency anemia in children under 5 years in a randomized controlled trial. The study reported hemoglobin changes and anemia correction rates, with tolerability data on adverse events [58]. While specific numerical results were not fully detailed, the study contributed to the growing evidence base for liposomal iron in pediatric populations.

Sawires et al. [59] conducted a cross-over study comparing oral liposomal iron with oral iron polymaltose in children with chronic kidney disease iron deficiency anemia. The study reported hemoglobin and ferritin changes and detailed tolerability and absorption insights [59]. The cross-over design allowed each patient to serve as their own control, strengthening the comparison.

Mahmoud [60] compared liposomal SunActive iron (a novel ferric pyrophosphate formulation) with conventional oral iron (iron polymaltose complex) in children aged 2–12 years for treatment of iron deficiency anemia. After 1 month, liposomal SunActive iron led to higher hemoglobin, hematocrit, serum ferritin, and serum iron levels, and greater transferrin saturation compared to conventional iron [60]. After 6 months, hemoglobin (*p* < 0.001) and iron profile (*p* < 0.001) were higher in the liposomal group. The study also showed reduced drug refusal rates and improved compliance with liposomal iron.

### 11.3. Clinical Trials and Efficacy Data

#### 11.3.1. Pregnancy and Women’s Health

Pregnancy represents a state of increased iron requirements due to expanded maternal blood volume, fetal development, and placental growth. Iron deficiency anemia in pregnancy is associated with adverse maternal and fetal outcomes, including preterm delivery, low birth weight, and postpartum hemorrhage.

Duarte et al. [61] reviewed oral iron supplementation in pregnancy, examining current recommendations and evidence-based medicine. The review included analysis of the Parisi et al. [62] randomized controlled trial, which compared liposomal iron formulations at 14 mg/day (LI14) and 28 mg/day (LI28) with 30 mg/day ferrous iron and placebo control in pregnant women. Daily iron supplementation significantly increased maternal hemoglobin by 4.59 g/L (95% CI: 3.72–5.46) and reduced anemia risk by 50–70% (RR 0.30–0.50) [61]. It also reduced iron deficiency (RR 0.33–0.59). Supplementation resulted in higher side effects (22% vs. 18%) but no statistical difference. Hemoglobin decreased in all groups in one study, but less pronouncedly with liposomal iron.

A comparison study of liposomal iron versus iron fumarate in pregnant women with mild anemia [63] demonstrated that the liposomal iron group showed significantly higher hemoglobin levels (11.18 ± 0.44) at 28–32 weeks compared to the ferrous iron group (10.70 ± 0.39). Liposomal iron dramatically raised hemoglobin and ferritin levels and was associated with reduced exposure to gastric contents and decreased interaction with food, suggesting improved tolerability without notable negative consequences [63].

#### 11.3.2. Chronic Kidney Disease

Chronic kidney disease (CKD) patients frequently develop iron deficiency due to reduced dietary intake, impaired intestinal absorption, chronic blood loss (gastrointestinal bleeding, hemodialysis), and inflammation-induced hepcidin elevation. Iron deficiency contributes to anemia in CKD and may limit the efficacy of erythropoiesis-stimulating agents.

Visciano et al. [64] proposed liposomal iron as a new treatment option for anemia in chronic kidney disease. In a randomized trial comparing intravenous iron with liposomal iron in CKD stages 3, 4, and 5 patients (1:2 ratio), the primary outcome of hemoglobin increase at 8 weeks was similar in both groups but statistically significant only in the liposomal group [64]. Secondary outcomes included a 25% erythropoietin dosage reduction and a 100 ng/mL serum ferritin increase, suggesting that liposomal iron may reduce the need for erythropoiesis-stimulating agents.

Maladkar et al. [12] reviewed the novel approach for iron deficiency anemia with liposomal iron from concept to clinic, including data from CKD patients. In one study, oral liposomal iron (30 mg/day) was compared to intravenous ferrous gluconate (1000 mg/day) in CKD patients. Oral liposomal iron was as efficacious as intravenous iron for hemoglobin increase, with significantly lower adverse events (3.1% vs. 34.5%, *p* < 0.001) [12].

#### 11.3.3. Pediatric Populations

Children are particularly vulnerable to iron deficiency due to rapid growth, increased iron requirements, and often limited dietary iron intake. Liposomal iron offers potential advantages in pediatric populations due to improved palatability and reduced gastrointestinal side effects.

Monazah et al. [55] demonstrated superior efficacy of liposomal iron versus ferrous sulfate in infants aged 4–6 months with iron deficiency anemia, as discussed in Section 11.2.1. The study’s findings are particularly important given that infancy represents a critical period for neurodevelopment, and iron deficiency during this period can have long-lasting cognitive and behavioral consequences.

Koura et al. [14] showed dramatic effects of liposomal iron on chronic kidney disease pediatric anemia, with efficacy comparable to or exceeding intravenous iron dextran and superior safety profile, as discussed in Section 11.2.2.

Mahmoud [60] demonstrated that liposomal SunActive iron was superior to conventional iron polymaltose complex in children aged 2–12 years, with better hematological outcomes and improved compliance due to reduced drug refusal rates.

#### 11.3.4. Special Populations and Conditions

Inflammatory Bowel Disease: Patients with inflammatory bowel disease (IBD) frequently develop iron deficiency due to chronic intestinal blood loss, malabsorption, and inflammation-induced hepcidin elevation. Tinsley et al. [54] noted that in inflammatory bowel disease patients, 62% of anemic patients increased hemoglobin or normalized levels with liposomal iron, improving quality of life and reducing fatigue.

Preoperative Optimization: Albanese et al. [65] compared ferric carboxymaltose and liposomal iron (SiderAL Forte) for enhancing preoperative iron supplementation in lipoabdominoplasty patients. The study aimed to optimize preoperative iron levels and prevent postoperative anemia, reporting hemoglobin and ferritin changes and tolerability details [65].

Animal Models: Szudzik et al. [66] demonstrated that innovative oral sucrosomial ferric pyrophosphate-based supplementation (6 mg Fe daily from day 5 to 28 of life) rescued suckling piglets from iron deficiency anemia similarly to commonly used parenteral therapy with iron dextran (100 mg Fe/kg body weight on day 3 of life). Oral sucrosomial iron-supplemented piglets showed significantly lower (*p* ≤ 0.05) plasma 8-isoprostane levels, indicating lesser toxicity [66]. Plasma iron levels were almost 2-fold higher (*p* < 0.05) with sucrosomial iron, and hepcidin levels were not considerably increased, supporting the mechanism of hepcidin-independent absorption.

### 11.4. Safety and Tolerability Profile

A consistent finding across multiple studies is the superior gastrointestinal tolerability of liposomal iron compared to conventional oral iron formulations. The mechanisms underlying improved tolerability include:

- Reduced gastric irritation: The phospholipid bilayer protects the gastric mucosa from direct iron contact, reducing nausea, epigastric discomfort, and gastritis [12].
- Lower oxidative stress: Conventional iron salts can generate reactive oxygen species in the gastrointestinal tract through Fenton chemistry, contributing to mucosal damage and symptoms. Liposomal encapsulation reduces this oxidative stress [66].
- Reduced constipation: Liposomal iron appears to cause less constipation than ferrous sulfate, possibly due to reduced unabsorbed iron in the colon [56].
- Improved taste: Liposomal formulations can be flavored and have better palatability than conventional iron syrups, improving acceptance particularly in pediatric populations [57,60].

Serious adverse events with liposomal iron are rare. Unlike intravenous iron, liposomal iron does not carry risks of infusion reactions, anaphylaxis, or iron overload from rapid administration. The gradual absorption profile of oral liposomal iron provides a safety margin against acute iron toxicity.

Despite these advantages, liposomal iron formulations have important limitations that require acknowledgement. Clinical trial data in iron deficiency anemia demonstrate that liposomal iron achieves smaller absolute hemoglobin increments compared to intravenous iron (e.g., 4.78% vs. 14.65% Hb increase reported by Zahr et al. [13]) and replenishes iron stores more slowly. Endocrine-specific randomized controlled trial data for liposomal iron are almost entirely absent, and most evidence discussed in this review is extrapolated from hematological endpoints in non-endocrine populations. Furthermore, the phospholipid encapsulation technology confers a substantially higher per-dose cost compared to standard ferrous sulfate (see Section 11.5), which may limit accessibility in resource-constrained settings or where reimbursement is unavailable.

### 11.5. Cost-Effectiveness Considerations

While liposomal iron formulations are typically more expensive per dose than generic ferrous sulfate, cost-effectiveness analyses must consider:

- Improved adherence: Higher adherence rates with liposomal iron may result in better clinical outcomes and reduced need for intravenous iron or blood transfusions [12].
- Reduced healthcare utilization: Fewer gastrointestinal side effects may reduce clinic visits, emergency department visits, and medication discontinuations.
- Comparison with intravenous iron: Liposomal iron is substantially less expensive than intravenous iron formulations when considering drug costs, administration costs, and monitoring requirements [11].
- Quality of life: Improved tolerability and convenience may provide quality-of-life benefits that are not captured in traditional cost-effectiveness analyses.
- Insurance coverage and cost: Liposomal iron is not uniformly covered by public or private health insurance systems in most countries. The per-dose cost is estimated to be 10–30-fold higher than generic ferrous sulfate, which may represent a significant barrier to long-term adherence for patients without comprehensive pharmaceutical coverage, particularly in the context of chronic endocrine diseases requiring prolonged supplementation.

Formal cost-effectiveness studies comparing liposomal iron with conventional oral and intravenous formulations across different clinical populations would be valuable for informing formulary decisions and treatment guidelines.

## 12. Discussion

This comprehensive review has examined the multifaceted relationships between iron deficiency and endocrine function across all major endocrine organ systems, revealing that iron plays far more extensive roles in endocrine physiology than traditionally recognized. The emerging field of “ferrocrinology” emphasizes that iron is not merely essential for hemoglobin synthesis but serves as a critical cofactor for numerous enzymes involved in hormone synthesis, metabolism, and cellular energy production [1].

### 12.1. Synthesis of Iron-Endocrine Relationships

Several unifying themes emerge from the evidence reviewed:

Enzymatic dependence: Multiple endocrine organs depend on iron-containing enzymes for hormone synthesis. Thyroid peroxidase (thyroid hormones) [2], cytochrome P450 enzymes (steroid hormones) [17], and tyrosine hydroxylase (catecholamines) [18] all require iron for catalytic activity. Iron deficiency therefore has the potential to impair hormone synthesis across multiple endocrine axes simultaneously.

Mitochondrial dysfunction: Iron is essential for mitochondrial oxidative phosphorylation through its roles in iron-sulfur clusters and heme groups in electron transport chain complexes [19]. Endocrine tissues have high metabolic demands, and iron deficiency-induced mitochondrial dysfunction may represent a common mechanism underlying endocrine dysfunction across multiple organ systems.

Bidirectional relationships: The relationships between iron status and endocrine function are bidirectional. Iron deficiency impairs endocrine function, but endocrine disorders can also predispose to iron deficiency through mechanisms including altered absorption (hypothyroidism) [29], increased losses (PMOS, hyperprolactinemia) [8], and dysregulated iron metabolism (obesity, diabetes) [6,31].

Inflammation and hepcidin: Chronic inflammation, characteristic of obesity, diabetes, and other metabolic disorders, leads to increased hepcidin production and functional iron deficiency [6,7]. This represents a form of iron deficiency distinct from nutritional iron deficiency and may require different therapeutic approaches.

The evidence base underpinning iron-endocrine interactions varies substantially in quality and mechanistic specificity across the organ systems reviewed. For thyroid function, a reasonable body of observational and interventional data supports clinically relevant associations [1,2,3,4]. For type 2 diabetes and polyendocrine metabolic ovarian syndrome (PMOS), evidence is primarily observational with limited randomized controlled trial data. For adrenal, parathyroid, pituitary, and pancreatic exocrine disorders, current evidence is largely mechanistic and hypothesis-driven, derived principally from animal models, case reports, and indirect clinical observations. Readers are cautioned against applying iron supplementation strategies to adrenal or parathyroid disorders based solely on the mechanistic arguments presented in this review; robust clinical evidence from adequately powered randomized controlled trials in these specific conditions is currently lacking.

### 12.2. Clinical Implications for Diagnosis

The recognition of iron-endocrine interactions has important implications for clinical diagnosis:

Screening recommendations: Iron status assessment should be considered in patients with endocrine disorders, particularly those with: (1) suboptimal response to hormone replacement therapy; (2) unexplained fatigue or other symptoms disproportionate to hormone levels; (3) concurrent anemia; (4) risk factors for iron deficiency (menorrhagia, vegetarian diet, malabsorption, chronic disease).

Interpretation challenges: Standard iron parameters may be difficult to interpret in patients with endocrine disorders and concurrent inflammation. Ferritin, an acute-phase reactant, may be falsely elevated in inflammatory states, masking true iron deficiency [7]. In such cases, additional markers including transferrin saturation, soluble transferrin receptor, and the sTfR/log ferritin ratio should be considered.

HbA1c accuracy: In diabetic patients with iron deficiency anemia, HbA1c may overestimate glycemic control due to prolonged erythrocyte lifespan [30,33]. Alternative glycemic monitoring methods (fasting glucose, continuous glucose monitoring) should be used until iron deficiency is corrected.

Thyroid function testing: Iron deficiency may affect thyroid function test results, potentially leading to misdiagnosis or inappropriate treatment adjustments [2,3]. Iron status should be assessed before attributing abnormal thyroid function tests to primary thyroid disease.

### 12.3. Therapeutic Considerations

The therapeutic approach to iron deficiency in patients with endocrine disorders requires consideration of several factors:

Timing of intervention: In patients with concurrent iron deficiency and endocrine dysfunction, iron repletion should generally be prioritized, as correction of iron deficiency may improve endocrine function and reduce the need for hormone replacement or adjustment [3,46].

Formulation selection: The choice of iron formulation should consider: (1) severity of iron deficiency; (2) presence of inflammation and hepcidin elevation; (3) gastrointestinal tolerability; (4) patient preference and adherence; (5) cost and accessibility.

Liposomal iron advantages: Liposomal iron formulations offer several advantages in patients with endocrine disorders: (1) superior gastrointestinal tolerability, important in patients with hypothyroidism-related constipation or diabetes-related gastroparesis [12]; (2) hepcidin-independent absorption pathways, beneficial in obesity and inflammatory conditions [11,12]; (3) comparable efficacy to intravenous iron in many populations [11,14]; (4) improved adherence due to better tolerability [56,60].

Monitoring: Response to iron supplementation should be monitored with: (1) hemoglobin and hematocrit at 4–8 weeks; (2) ferritin and transferrin saturation at 8–12 weeks; (3) reassessment of endocrine function after iron repletion; (4) evaluation of symptom improvement.

### 12.4. Gaps in Knowledge and Future Research Directions

Despite the growing evidence base, several important knowledge gaps remain:

Mechanistic studies: Further research is needed to fully elucidate the molecular mechanisms linking iron deficiency to endocrine dysfunction, including: (1) effects on specific enzyme activities; (2) gene expression changes mediated by iron-responsive elements; (3) epigenetic modifications related to iron status; (4) tissue-specific iron requirements and regulation.

Dose-response relationships: The optimal iron status for endocrine function remains unclear. While severe iron deficiency clearly impairs endocrine function, the effects of marginal iron deficiency and the optimal ferritin targets for different endocrine disorders require further investigation.

Intervention trials: Randomized controlled trials are needed to determine whether iron supplementation improves endocrine outcomes in patients with concurrent iron deficiency and endocrine disorders. Specific populations of interest include: (1) hypothyroid patients with suboptimal levothyroxine response; (2) diabetic patients with insulin resistance; (3) PMOS patients with anovulation; (4) men with hypogonadism; (5) children with growth retardation.

Liposomal iron in endocrine disorders: While liposomal iron has been extensively studied in general populations with iron deficiency anemia, specific studies in patients with endocrine disorders are limited. Research is needed to determine whether liposomal iron offers particular advantages in these populations.

Comparative effectiveness: Head-to-head trials comparing different iron formulations (oral conventional, oral liposomal, intravenous) in patients with specific endocrine disorders would inform evidence-based treatment guidelines.

Long-term outcomes: Most studies of iron supplementation focus on short-term hematological outcomes. Research examining long-term effects on endocrine function, quality of life, and clinical outcomes (e.g., fertility, cardiovascular events, diabetes complications) would be valuable.

Personalized approaches: Investigation of factors that predict response to different iron formulations (genetic polymorphisms, baseline hepcidin levels, inflammatory markers, microbiome composition) could enable personalized iron supplementation strategies.

### 12.5. Limitations

This review has several limitations that should be acknowledged:

Heterogeneity of evidence: The quality and quantity of evidence vary substantially across different endocrine disorders. While the thyroid-iron relationship is supported by systematic reviews and meta-analyses [2], evidence for other endocrine systems relies more heavily on observational studies and mechanistic research.

Publication bias: Studies showing positive associations between iron deficiency and endocrine dysfunction may be more likely to be published than null studies, potentially overestimating the strength of these relationships.

Confounding: Many studies examining iron-endocrine relationships are observational and subject to confounding by factors such as diet, inflammation, chronic disease, and socioeconomic status.

Causality: While bidirectional relationships between iron status and endocrine function are biologically plausible and supported by mechanistic studies, establishing causality requires intervention trials, which are limited for many endocrine disorders.

Generalizability: Most studies have been conducted in specific populations (e.g., pregnant women, children, CKD patients), and findings may not generalize to other populations.

## 13. Conclusions

Iron deficiency represents a common and underrecognized contributor to endocrine dysfunction across multiple organ systems. This comprehensive review has demonstrated that iron plays essential roles in thyroid hormone synthesis, insulin secretion and sensitivity, steroid hormone production, catecholamine synthesis, and overall endocrine function through its roles in iron-dependent enzymes and mitochondrial energy metabolism.

Key conclusions include:

- Thyroid function: Iron deficiency impairs thyroid hormone synthesis through reduced thyroid peroxidase activity and is associated with lower TSH, FT4, and FT3 levels, as well as increased thyroid autoantibody positivity [2,21]. Iron supplementation may improve thyroid function in patients with subclinical hypothyroidism and concurrent iron deficiency [3].
- Diabetes mellitus: Iron deficiency affects both insulin synthesis and sensitivity, with complex relationships to type 1 and type 2 diabetes [1,4,5]. Iron deficiency anemia falsely elevates HbA1c values, potentially leading to inappropriate treatment intensification [30,33].
- Obesity: Obesity-related chronic inflammation increases hepcidin production, leading to functional iron deficiency despite adequate iron stores [6,7]. This iron sequestration contributes to metabolic dysfunction and may increase type 2 diabetes risk [1,31].
- PMOS: Women with PMOS have increased risk of iron deficiency due to menstrual blood loss, inflammation, and obesity [8]. Iron deficiency may exacerbate anovulation, insulin resistance, and fertility impairment in PMOS patients.
- Male hypogonadism: Iron deficiency impairs testosterone synthesis through effects on cytochrome P450 enzymes in Leydig cells and may affect spermatogenesis and sperm quality [9]. Iron repletion should be considered before initiating testosterone replacement therapy in iron-deficient men.
- Other endocrine systems: Iron deficiency may affect adrenal function (cortisol and catecholamine synthesis) [1,18], pituitary function (growth hormone-IGF-1 axis) [46], and potentially parathyroid function and calcium metabolism [50].
- Liposomal iron: Liposomal iron formulations offer favorable gastrointestinal tolerability compared to conventional oral iron, with efficacy comparable to intravenous iron in many populations; however, IV iron remains superior for rapid iron store replenishment [11,12,14,55]. The hepcidin-independent absorption pathways of liposomal iron may offer particular advantages in inflammatory conditions [11,12].
- Clinical recommendations: Iron status should be routinely assessed in patients with endocrine disorders, particularly those with suboptimal treatment response or unexplained symptoms. Iron deficiency should be corrected using appropriate formulations, with liposomal iron representing an attractive option due to favorable tolerability and adherence.

In conclusion, the recognition of iron as a critical factor in endocrine physiology—the concept of “ferrocrinology”—has important implications for clinical practice. Clinicians managing patients with endocrine disorders should maintain a high index of suspicion for concurrent iron deficiency, assess iron status appropriately, and implement evidence-based iron supplementation strategies. Liposomal iron formulations represent a significant advance in iron supplementation, offering improved tolerability and efficacy compared to conventional oral iron while maintaining advantages of oral administration over intravenous routes. Future research should focus on intervention trials examining whether iron supplementation improves endocrine outcomes, comparative effectiveness studies of different iron formulations in endocrine populations, and mechanistic studies elucidating the molecular basis of iron-endocrine interactions. By integrating knowledge of iron-endocrine relationships into clinical practice, we can optimize diagnostic accuracy, therapeutic efficacy, and patient outcomes in this increasingly recognized clinical scenario (Table 1).

## Figures and Tables

**Table 1:** Summary of Major Clinical Studies on Iron Deficiency in Endocrine Diseases and Liposomal Iron Supplementation.

Author/Year	Study Design	Population	Iron Parameter	Endocrine Outcome	Key Finding
Iron Deficiency in Endocrine Diseases					
Garofalo et al. 2023 [2]	Systematic review and meta-analysis (10 cross-sectional studies)	Patients with iron deficiency vs. healthy controls	Serum ferritin, hemoglobin	TSH, FT4, FT3, thyroid autoantibodies	Iron deficiency associated with lower TSH (MD: −0.24 mIU/L, *p* = 0.005), FT4 (MD: −1.18 pmol/L, *p* < 0.000001), and FT3 (MD: −0.22 pmol/L, *p* < 0.00001); increased thyroid autoantibody positivity
Islam et al. 2021 [21]	Cross-sectional study	452 anemic women (0–81+ years), Bangladesh	Serum iron, TIBC, ferritin	TSH, FT4, hypothyroidism prevalence	24.69% of IDA patients had hypothyroidism (peak 38.46% in 31–40 years); strong correlation between iron and TSH (*p* < 0.0001); 50% of children with congenital hypothyroidism had IDA
Ravanbod et al. 2013 [3]	Intervention study	Patients with subclinical hypothyroidism and IDA	Hemoglobin, ferritin	Thyroid function parameters	Iron supplementation improved thyroid function in patients with subclinical hypothyroidism and concurrent iron deficiency Treatment: ferrous sulfate supplementation; dose: 65 mg/day (elemental iron); duration: 3 months
Christy et al. 2014 [30]	Cross-sectional study	Diabetic individuals with controlled glucose	Hemoglobin, iron status	HbA1c levels	IDA associated with significantly elevated HbA1c despite similar fasting and postprandial glucose; iron deficiency prolongs erythrocyte lifespan, falsely elevating HbA1c
Fernández-Real et al. 2002 [5]	Review	Patients with diabetes and iron metabolism disorders	Serum ferritin, iron indices	Insulin resistance, diabetes risk	Elevated body iron stores associated with increased T2DM risk; iron-induced oxidative stress impairs insulin signaling and β-cell function
Szklarz et al. 2022 [1]	Narrative review	General population	Iron deficiency	Multiple endocrine systems	Iron deficiency impairs exercise capacity, reduces AMPK activity, enhances insulin resistance, causes brown adipose tissue dysfunction, impairs thyroid hormone binding, and affects cortisol synthesis
Cepeda-Lopez et al. 2010 [6]	Review	Obese individuals	Serum iron, ferritin, hepcidin	Obesity-related iron deficiency	Obesity increases iron deficiency risk 2–3 fold through chronic inflammation and hepcidin-mediated iron sequestration
Al-Akabi et al. 2023 [8]	Cross-sectional study	Women with PMOS vs. controls, Basrah	Serum iron, transferrin saturation, TIBC	PMOS features	PMOS patients had lower serum iron, reduced transferrin saturation, and higher TIBC; increased iron deficiency prevalence in PMOS
Prasad et al. 1961 [9]	Clinical case series	Young men with severe iron deficiency	Hemoglobin, iron status	Testosterone, testicular size, sexual maturation	Severe iron deficiency associated with hypogonadism, delayed sexual maturation, reduced testicular size; iron supplementation improved testosterone and sexual development Treatment: iron and zinc sulfate supplementation; dose/duration not specified in original report [9].
Alkuwari et al. 2025 [46]	Systematic review (includes animal models)	Children with IDA	Hemoglobin, ferritin	Growth velocity, pubertal development	IDA associated with growth retardation and delayed puberty; iron supplementation improved growth velocity and normalized endocrine function Treatment: oral iron supplementation; dose/duration as reported in conference abstract [46].
Liposomal Iron Supplementation					
Pisani et al. 2015 [11]	Randomized controlled trial	Non-dialysis CKD patients with IDA	Hemoglobin, ferritin, transferrin saturation	N/A	Oral liposomal iron (30 mg/day) vs. IV iron gluconate (1000 mg total): similar final Hb increase; IV showed greater iron store replenishment; oral had significantly lower adverse events (*p* < 0.001)
Zahr et al. 2024 [13]	Non-inferiority RCT	Non-dialysis CKD patients	Hemoglobin, ferritin, TSAT	N/A	Oral liposomal iron (30 mg/day) vs. IV iron sucrose: IV group 14.65% Hb increase (*p* = 0.049), oral 4.78% (*p* = 0.003); IV hypotension 30.7%, oral constipation 28.4%; oral safe and effective but less efficacious for iron stores
Koura et al. 2025 [14]	Randomized clinical trial	Pediatric CKD patients with anemia	Hemoglobin, ferritin	N/A	Oral liposomal iron (30 mg/day) vs. IV iron dextran (50 mg 3×/week): oral showed higher efficacy with no side effects and higher compliance; no significant difference between groups for Hb and ferritin improvements
Monazah et al. 2025 [55]	Randomized controlled trial	Infants 4–6 months with IDA	Hemoglobin, MCH, ferritin, MCV, TIBC	N/A	Liposomal iron (15 mg/mL) vs. ferrous sulfate (25 mg/mL) daily for 6 months: liposomal showed greater Hb increase (β = 0.35 at 2 months, β = 0.51 at 6 months, both *p* < 0.05) and MCH increase despite lower iron content
Rangaraj et al. 2025 [56]	Randomized controlled trial	Children 6–60 months with IDA	Hemoglobin	N/A	Liposomal iron (1 mg/kg/day) vs. ferrous ascorbate (3 mg/kg/day) for 1 month: ferrous ascorbate superior Hb increase (9.3 vs. 8.5 g/dL, *p* = 0.0018); liposomal better adherence (*p* = 0.047), tolerance (*p* = 0.042), less constipation (*p* = 0.046)
Hussain et al. 2019 [57]	Open-label clinical trial	558 women with IDA	Hemoglobin	N/A	Microencapsulated liposomal iron (14 mg/sachet, 2×/day) for 12 weeks: Hb increased from 8.71 ± 2.24 to 10.47 ± 1.69 g/dL (*p* < 0.001); enhanced palatability, higher bioavailability, reduced GI side effects; 21.6% dropout rate No comparator arm (single-arm open-label trial).
Mahmoud, 2025 [60]	Prospective RCT	Children 2–12 years with IDA	Hemoglobin, hematocrit, ferritin, serum iron, transferrin saturation	N/A	Liposomal SunActive iron vs. iron polymaltose for 1 and 6 months: liposomal showed higher Hb, hematocrit, ferritin, serum iron, and transferrin saturation at both timepoints (*p* < 0.001 at 6 months); reduced drug refusal, improved compliance
Maladkar et al. 2020 [12]	Narrative review	Multiple populations (pregnant women, CKD, menstruating women)	Hemoglobin, ferritin	N/A	Liposomal iron significantly increased Hb and ferritin in pregnant women (*p* < 0.01) and iron-deficient women (79% ferritin increase, *p* ≤ 0.001); better tolerability, fewer GI side effects; in CKD, oral liposomal as efficacious as IV with lower adverse events (3.1% vs. 34.5%, *p* < 0.001) Comparison: liposomal iron vs. oral ferrous sulfate (pregnant and menstruating women); vs. IV ferrous gluconate 1000 mg/day (CKD cohort).
Szudzik et al. 2020 [66]	Randomized trial (animal model)	Suckling piglets	Plasma iron, 8-isoprostane, hepcidin	N/A	Oral sucrosomial ferric pyrophosphate (6 mg Fe/day, days 5–28) vs. parenteral iron dextran (100 mg Fe/kg, day 3): comparable IDA correction; oral showed lower oxidative stress (lower 8-isoprostane, *p* ≤ 0.05), 2-fold higher plasma iron (*p* < 0.05), no hepcidin increase

Abbreviations: CKD, chronic kidney disease; FT3, free triiodothyronine; FT4, free thyroxine; GI, gastrointestinal; Hb, hemoglobin; HbA1c, hemoglobin A1c; IDA, iron deficiency anemia; IV, intravenous; MCH, mean corpuscular hemoglobin; MCV, mean corpuscular volume; MD, mean difference; PMOS, polyendocrine metabolic ovarian syndrome; RCT, randomized controlled trial; TIBC, total iron-binding capacity; TSH, thyroid-stimulating hormone; TSAT, transferrin saturation.

## Data Availability

No new data were created or analyzed in this study Data sharing is not applicable.
